# A new class of radiosensitizers for glioblastoma

**DOI:** 10.18632/oncotarget.27970

**Published:** 2021-06-22

**Authors:** Roger Abounader, David Schiff

**Keywords:** glioblastoma, radiation therapy, mibefradil

***News on:** Creation of a new class of radiosensitizers for glioblastoma based on the mibefradil pharmacophore by Paradkar et al. Oncotarget. 2021; 12:891–906. https://doi.org/10.18632/oncotarget.27933. [PubMed]*

Glioblastoma (GBM) is the most common and most deadly primary malignant brain tumor. Current standard of care consists of surgery followed by chemo- and radiotherapy. Almost all GBM patients recur and develop therapy resistance. Radiosensitization of GBM could partially overcome therapy resistance and improve outcomes. Radiation-induced tumor cytotoxicity is partly mediated by DNA damage. Consequently, inhibition of DNA repair can sensitize tumors to radiation. There is a long history of unsuccessful attempts to improve GBM outcome with radiosensitizers. The approach furthest along at present utilizes PARP inhbitiors, with the results of a Phase 3 trial adding veliparib to standard therapy (NCT02152982) anticipated shortly. Other ongoing efforts focus on inhibitors of Wee1, ATM, and ATR. An interesting and therapeutically consequential study published by Bindra and colleagues in this issue of Oncotarget describes the development and testing of a novel class of DNA repair inhibitors that could potentially be used as GBM radiosensitizers (Pradakar et al., Oncotarget, in this issue).

Initial studies in GBM of the calcium channel blocking drug mibefradil, originally developed as an antihypertensive, were based on its activity as a cell cycle synchronizer at the G1/S checkpoint and led to an Adult Brain Tumor Consortium (ABTC 1101) clinical trial of timed sequential therapy in conjunction with the alkylating agent temozolomide [1]. More recently, Bindra and colleagues discovered that mibefradil inhibits non-homologous end joining repair and sensitizes GBM to radiation therapy via a mechanism that does not involve calcium blockage. A phase I clinical trial of mibefradil in combination with radiation therapy showed some promising responses but also cardiac conduction adverse effects that the authors attributed to mibefradil’s calcium channel blocking activity. Furthermore, mibefradil’s utility as an antihypertensive was limited by numerous drug-drug interactions through inhibition of multiple cytochrome P450 enzymes, resulting in its withdrawal from the market. Consequently, they sought to minimize the adverse effects by creating a new class of radiosensitizers that retained mibefradil’s DNA repair inhibitor activity but had lesser channel blocking activity and P450 interaction. To that end, they used structure activity relationship analysis to create and synthesize 140 analogues of mibefradil. They profiled the analogs using a microplate-based assay that they had previously developed and published and that simultaneously measures homologous recombination and non-homologous end joining in live cells [2]. They found that the analogs were potent inhibitors of homologous recombination and non-homologous end joining with reduced calcium channel blocking activity. They tested the pharmacokinetic parameters of the synthesized analogues and confirmed their ability to cross the blood brain barrier and accumulate in mouse brain tissue at levels similar to those observed with mibefradil. They also found that the tetrahydronaphthalene core and tertiary amine in mibefradil contributed to its non-homologous end joining inhibitory function, while the benzimidazole ring and the methoxyacetate group (responsible for the calcium blockage ability) were non-essential.

The findings of the study describe a new class of mibefradil-based DNA repair inhibitors, which could eventually be tested as GBM radiosensitizers in preclinical and clinical trials. The use of DNA repair inhibitors as radiosensitizers in GBM is a promising approach to achieving better therapeutic responses owing to the range of repair pathways that are activated in response to radiation-induced DNA damage. One specific analog, YU252386, showed promise as a potent and selective radiosensitizer that showed a > 10-fold decrease in T-type channel activity as compared to mibefradil. The identification of the moieties responsible for inhibition of DNA repair vs. the ones responsible for calcium channel blockage could aid the development of more selective analogs that have potent DNA repair inhibitory activities and lesser calcium blocking activities and would presumably be better radiosensitizers with fewer adverse effects than mibefradil. However, the elimination of calcium blockage activity might not be desirable in all therapeutic settings. In fact, other studies have shown that calcium blockage with mibefradil has potent anti-tumor effects that are mediated by the inhibition of oncogenic pathways and transcriptional events that are unrelated to DNA damage repair [3]. Therefore, elimination of calcium channel blockage activity might reduce the anti-GBM effects of mibefradil-related compounds in certain settings. Additionally, the aforementioned ABTC 1101 trial of mibefradil with temozolomide found mibefradil to be well tolerated [1] with evidence of clinical activity, suggesting that the future use of mibefradil and other calcium channel blocker for GBM therapy might still be justified in certain contexts. Nonetheless, the compounds that were created in the study being discussed in this editorial offer new GBM therapeutic options that could be beneficial in certain settings that remain to be determined in the context of personalized medicine. However, before use in humans, a number of investigative studies need to be performed. These would include pharmacodynamics, efficacy, and toxicology studies in various representative animal models of GBM to determine if the compounds can inhibit tumor growth as single therapies and in combination with radiation therapy and temozolomide chemotherapy. The doses and timing of the compounds relative to radio-chemotherapy would have to be determined. Potential biomarker studies to identify factors that might predict sensitivity to the compounds could also be conducted. The data from these experiments would be used to inform subsequent clinical trials. The remarkable lack of progress since the introduction of fractionated radiotherapy in improving outcome from GBM emphasizes the urgent unmet need mibefradil-based radiation sensitizers could play.
